# RETRACTION: A Systematic Review of the Exercise Effects on Burn Wound Healing

**DOI:** 10.1111/iwj.70275

**Published:** 2025-03-03

## Abstract

**RETRACTION**: NiumanlanY. Jingming, , HaoQ. , FarzanR. , and OtaghvarH. A. , “A Systematic Review of the Exercise Effects on Burn Wound Healing,” International Wound Journal 21, no. 3 (2024): e14482, 10.1111/iwj.14482.37957133 PMC10898404

The above article, published online on 13 November 2023, in Wiley Online Library (http://onlinelibrary.wiley.com/), has been retracted by agreement between the journal Editor in Chief, Professor Keith Harding; and John Wiley & Sons Ltd. Following an investigation by the publisher, all parties have concluded that this article was accepted solely on the basis of a compromised peer review process. In addition, the publisher confirmed multiple instances of excessive self‐citations in the reference list of this article. In view of the clear evidence of compromised peer review, the parties agreed that the paper must be retracted. The authors disagree with the retraction.

**RETRACTION**: Niumanlan, Y. Jingming , Q. Hao , R. Farzan , and H. A. Otaghvar , “A Systematic Review of the Exercise Effects on Burn Wound Healing,” International Wound Journal 21, no. 3 (2024): e14482, 10.1111/iwj.14482.37957133 PMC10898404

The above article, published online on 13 November 2023, in Wiley Online Library (http://onlinelibrary.wiley.com/), has been retracted by agreement between the journal Editor in Chief, Professor Keith Harding; and John Wiley & Sons Ltd. Following an investigation by the publisher, all parties have concluded that this article was accepted solely on the basis of a compromised peer review process. In addition, the publisher confirmed multiple instances of excessive self‐citations in the reference list of this article. In view of the clear evidence of compromised peer review, the parties agreed that the paper must be retracted. The authors disagree with the retraction.

